# Depression as a concealable stigmatized identity: what influences whether students conceal or reveal their depression in undergraduate research experiences?

**DOI:** 10.1186/s40594-020-00216-5

**Published:** 2020-06-04

**Authors:** Katelyn M. Cooper, Logan E. Gin, Sara E. Brownell

**Affiliations:** 1 Department of Biology, University of Central Florida, 4110 Libra Dr., Orlando, FL 32816 USA; 2 Research for Inclusive STEM Education Center, Biology Education Research Lab, School of Life Sciences, Arizona State University, PO Box 874501, Tempe, AZ 85287-4501 USA

**Keywords:** Mental health, Depression, STEM, Life sciences, Biology, Concealable stigmatized identities, CSI, Stigma, Research, Undergraduate research experiences, Identity interference, Role model

## Abstract

**Background:**

Concealable stigmatized identities (CSIs) are identities that can be kept hidden or invisible and that carry negative stereotypes. Depression is one of the most common CSIs among undergraduates. However, to our knowledge, no studies have explored how students manage depression as a CSI in the context of undergraduate research, a high-impact practice for undergraduate science students. Concealing CSIs can cause psychological distress and revealing CSIs can be beneficial; however, it is unknown whether these findings extend to students with depression in the context of undergraduate research experiences. In this study, we interviewed 35 life sciences majors with depression from 12 research-intensive institutions across the United States who participated in undergraduate research. We sought to understand to what extent students reveal their depression in research and to describe the challenges of concealing depression and the benefits of revealing depression in this specific context. Additionally, we explored whether students knew scientists with depression and how knowing a scientist with depression might affect them.

**Results:**

Most students did not reveal their depression in their undergraduate research experiences. Those who did typically revealed it to another undergraduate researcher and few revealed it to a faculty mentor. Students who concealed their depression feared the potential consequences of revealing their identity, such as being treated negatively by others in the lab. Students who revealed their depression highlighted a set of benefits that they experienced after revealing their depression, such as receiving support and flexibility from their research mentor. We found that few students knew a specific scientist with depression. However, students perceived that knowing a scientist with depression would help them realize that they are not the only one experiencing depression in science and that people with depression can be successful in science.

**Conclusions:**

This study illustrates that students with depression would benefit from research environments that are supportive of students with depression so that they can feel comfortable revealing their depression if they would like to. We also identified that students may benefit from knowing successful scientists with depression. We hope this study encourages undergraduate research mentors to support students with depression and ultimately reduces the stigma around CSIs such as depression.

## Introduction

Undergraduate research experiences in science, technology, engineering, and math (STEM), or opportunities where students work in faculty members’ labs on a research project, are increasingly the critical gateway into a professional STEM career. STEM graduate and professional programs in the United States, particularly top programs, consider undergraduate research as a requirement for entry (Cooper et al. 2019c; Olson and Riordan 2012). Therefore, identifying how we can more equitably engage students in undergraduate research and maximize the participation of students in undergraduate research is foundational for diversifying the scientific community.

Efforts to document and maximize the experiences of underrepresented and underserved students in undergraduate research have primarily focused on identities that are conspicuous or that tend to be immediately visible, such as gender and race/ethnicity^1^ (Byars-Winston et al. 2015; Glenn et al. 2012; Grineski et al. 2018; Harsh et al. 2012; K. A. Kim et al. 2011). There is very limited research on the experiences of students with invisible identities in this unique context, although a few studies have documented the experiences of first-generation college students and students from low socioeconomic backgrounds (Cooper et al. 2019b; Eagan Jr et al. 2013; Ishiyama 2007; Y. K. Kim and Sax 2009; Mekolichick and Gibbs 2012). We argue that studying the experiences of students with concealable identities, and specifically concealable stigmatized identities (CSIs), in undergraduate research is a ripe area of research with the potential to advance knowledge about how to effectively create more inclusive research experiences.

This research explores the experiences of students with depression, one of the most common concealable stigmatized identities (Chaudoir and Quinn 2010), in the context of undergraduate research. We use the frame of concealable stigmatized identities to understand what influences students to reveal or conceal their depression in the context of undergraduate research, to understand the benefits of revealing depression in research, and to identify the extent to which students with depression have found role models with depression in the scientific research community.

### Background

#### Concealable stigmatized identities

A concealable stigmatized identity, or CSI, is an identity that can be kept hidden or concealed from others and that has negative attributes or stereotypes attached, which can result in a loss of status and/or discrimination in society (Link and Phelan 2001; Quinn and Earnshaw 2011). The extent to which an identity is stigmatized can vary across cultures and specific situations. In the United States (U.S.), for example, CSIs include but are not limited to mental illness, certain medical conditions such as AIDS, coming from a low socioeconomic background, and being a member of the lesbian, gay, bisexual, transgender, and queer (LGBTQ+) community (Chaudoir and Quinn 2010; Kallschmidt and Eaton 2019).

It is well established that having and concealing a stigmatized identity can lead to increased psychological distress. Researchers have found that psychological distress is most severe (a) when individuals anticipate stigma or worry what will happen when others learn about their CSI, (b) when someone considers their CSI to be a central part of who they are, (c) for those who frequently think about their CSI, and (d) for individuals with CSIs that are especially culturally stigmatized (Quinn et al. 2014; Quinn and Chaudoir 2009). People with CSIs often develop internalized stigma because many CSIs, such as depression, are realized later in life once individuals have learned negative stereotypes about their identity (Link 1987; Quinn and Earnshaw 2011). One can learn stereotypes about CSIs from the media, for example, one study showed that mental illness is commonly depicted in children’s television shows, but the depiction is often negative, showing those with mental illness as unattractive, violent, and criminal (Wahl 2003). Stereotypes can also be learned from family and friends who may speak negatively about particular stigmatized identities, especially before they know that a family member or friend has a particular CSI (Dovidio et al. 2010). Internalized stigma can negatively affect a person’s well-being because it is a significant contributor to psychological stress (Mak et al. 2007), and this has been demonstrated specifically in people with mental health CSIs (Ritsher and Phelan 2004).

#### Concealable stigmatized identities in research

Understanding the experiences of students with CSIs in undergraduate research is an important step toward creating more inclusive research experiences with the potential to improve the retention of underserved undergraduates in STEM. Specifically, we hypothesize that both the concealable nature of one’s identity and the stigmatization of that identity can present unique challenges for students that warrant further attention from both education researchers and research mentors who can affect students’ research experiences. Identity management, or the decisions about when, how, where, and to whom to disclose CSIs, and the decisions associated with identity management can result in strain and stress for individuals (Goffman 2009; Jones and King 2014). Students who feel as though they need to conceal their CSI in their research environment likely experience greater levels of stress and anxiety, since concealing one’s CSI can lead to psychological distress (Quinn et al. 2014). Navigating one’s CSI in the context of undergraduate research may be especially distressing if an undergraduate experiences identity interference, defined as their CSI interfering with their identity as an undergraduate researcher (National Academies of Sciences, Engineering, and Medicine 2019). As an identity management strategy, students who experience identity inference may choose to compartmentalize their CSI and their identity as an undergraduate researcher; this compartmentalization can further students’ stress if they feel that they cannot authentically be who they are in their research experience (Malone and Barabino 2009; McCoy et al. 2015; Puckett et al. 2016; Tate and Linn 2005; Yoder and Mattheis 2016).

The disclosure process model is a framework that examines when and why disclosing identities can be beneficial and can help explain whether students choose to reveal their CSI in the context of research (Chaudoir and Fisher 2010). The disclosure process model suggests that when individuals disclose information to others about their CSI, it affects their lives in three ways: (1) it can provide alleviation of inhibition; in other words, disclosing one’s CSI can alleviate the psychological and physiological stress caused by inhibition, (2) it can change their social support or make the person disclosing their identity vulnerable to social evaluation that can either result in greater social support or greater stigmatization, and (3) it can change their social information; that is, once one’s CSI is “out in the open”, it can change the perceptions and actions of both the confidant and the discloser. Therefore, the disclosure process model may help explain whether individuals choose to reveal or conceal their CSI, specifically, an undergraduates’ social support structure and their perception of what others might do after learning about their CSI may influence their decision. Learning to manage their identity in the context of research is just one challenge that undergraduates with CSIs may face.

Identifying effective research mentors for students with CSIs could be uniquely challenging, yet especially critical to students’ success in research. Effective mentorship includes providing students with psychosocial support, which has been shown to increase the chance of recruitment and retention in STEM careers (Dodson et al. 2009; National Academies of Sciences, Engineering, and Medicine 2019; Wendt et al. 2018). Psychosocial support includes role modeling and providing encouragement and counseling, which can help students cope with stressors in research (G. Crisp and Cruz 2009; National Academies of Sciences, Engineering, and Medicine 2019). Although psychosocial support has been found to be important for individuals with visible stigmatized identities, it has been said to be vital for those with CSIs since these individuals may have fewer opportunities for social support and validation compared to those with visible stigmas (Deaux and Ethier 1998; Miller 2006; Miller and Major 2000).

Mentoring experts suggest that “effective mentorship requires that faculty have an awareness of identity-related challenges their mentees have” (National Academies of Sciences, Engineering, and Medicine 2019, p. 60). If students with CSIs conceal their identities, then it is difficult, if not impossible, for research mentors to recognize and address identity-related challenges. Further, some studies suggest that students prefer and can benefit from mentors who share their same identity, especially if their identities are stigmatized; so far, this has been demonstrated with gender and race/ethnicity (Blake-Beard et al. 2011; Dee 2004; Patton and Bondi 2015). Because both students and potential research mentors are unlikely to reveal their CSIs in professional settings (Cooper et al. 2019a; Cooper and Brownell 2016; Ridge et al. 2019), finding a mentor with the same CSI is likely extremely difficult; and to our knowledge, there is little research about whether same-identity mentoring is effective for students with CSIs in the context of undergraduate research.

Identifying role models or scientists with CSIs whom students can look up to may be uniquely beneficial for undergraduate researchers with CSIs. Studies have shown that people with CSIs in the sciences can serve as important role models to students, for example, research shows that LGBTQ+ instructors can serve as positive role models for LGBTQ+ students, highlighting that LGBTQ+ people can be successful in science (Cooper et al. 2019a; Cooper and Brownell 2016). In sum, undergraduate research may present unique challenges for students with CSIs, owing to the concealable nature and stigmatization of their identity; specifically, students with CSIs may experience additional stress and anxiety if they conceal their identity in the context of research, find it difficult to locate effective mentors whom they trust to help them mitigate identity-related challenges, and struggle to identify public role models in science who share their CSIs.

#### Depression, one of the most common CSIs

In this study, we investigate how students with depression manage their CSI in the context of undergraduate research. Depression is a common and serious mood disorder that results in persistent feelings of sadness and hopelessness, as well as, a loss of interest in activities that one once enjoyed (American Psychiatric Association 2013). We chose to focus this study on depression because, in the U.S., mental illness is the most common CSI among college students (Chaudoir and Quinn 2010) and depression, specifically, is thought to affect approximately 35% of college science students (Gin LE, Cooper KM, Brownell SE: Student identities in undergraduate biology, unpublished; Ibrahim et al. 2013). Further, depression is one of the primary concerns among U.S. college and university counseling centers because of the detrimental impact it can have on undergraduates (Center for Collegiate Mental Health 2017). Physiology and stressors both play an important role in the etiology of depression (Saveanu and Nemeroff 2012). Specifically, the heightened stress of college is known to contribute to student depression (Dyson and Renk 2006). College students report that depression can negatively affect their grades and can contribute to their inability to complete a course (American College Health Association 2019). Further, studies have shown that students with mental illness CSIs, such as anxiety and depression, experience stereotype threat in academic settings (Quinn et al. 2004). That is because there can be a stereotype that people with mental illness struggle academically, when people with mental illness are in a situation where they believe their academic ability is being evaluated, and their identity is relevant, they tend to underperform compared to their counterparts without mental illnesses. However, this is not true when students do not perceive their CSI to be relevant to the task (Quinn et al. 2004). Despite the ubiquity of depression among college students and evidence suggesting that students with depression may have a particularly difficult time in undergraduate research (Cooper et al. 2020), no studies have explored how students with depression navigate their CSI in the context of undergraduate research. Understanding more about how students with depression manage their CSIs in this unique context can provide insight into how the scientific research community can create more inclusive research environments for students with depression.

### Current study

We conducted qualitative interviews with a national sample of students with depression in the United States who had participated in undergraduate research. We organized the research study into two parts, the first of which focuses on the effects of revealing or concealing one’s depression in research, and the second of which documents students’ awareness of potential role models with depression in the sciences.

Our research questions for part 1 of the study were:

- To what extent do students reveal their depression in undergraduate research and why?
- What are the potential benefits of revealing depression and the challenges of concealing depression in the context of undergraduate research?

Our research questions for part 2 of the study were:

- To what extent do students know scientists who have depression?
- How are students affected by knowing a scientist with depression or hypothetically how do students perceive they would be affected by knowing a scientist with depression?

## Methodology

### Interview recruitment

In spring 2019, we emailed 496 undergraduate researchers from 25 research-intensive (R1) institutions who had previously participated in a national survey about their undergraduate research experience and indicated that they would be interested in participating in a follow-up interview about their research experience. The interview recruitment email explicitly stated that we were recruiting students who self-identified as having depression because we were interested in learning more about the experiences of students with depression in undergraduate research. In the U.S., mental healthcare is disproportionately unavailable to Black and Latinx individuals, as well as individuals who come from low socioeconomic backgrounds (Howell and McFeeters 2008; Kataoka et al. 2002; Santiago et al. 2013); as such, to prevent a biased sample, we explicitly recruited students who self-identified as having depression and made it clear that we did not require students to be formally diagnosed with depression to participate in the study. Students were offered a $15 gift card as an incentive to participate in the interview and 35 students, representing 12 of the 25 R1 institutions, agreed to participate in the study.

### Interviews

We developed an interview script to probe whether students revealed or concealed their CSI (depression) and the reasoning behind their decision. We also explored students’ perceptions of the potential benefits and challenges of revealing their identity to members of their lab including other undergraduates, graduate students, postdocs, or faculty who supervised or guided their research. Additionally, we asked students whether they knew any scientists with depression and how knowing or potentially knowing a scientist with depression might affect them. Once the interview script was developed, we conducted a series of think-aloud interviews in order to establish cognitive validity of the questions (Trenor et al. 2011) and to screen for any question that might make undergraduates uncomfortable due to the sensitive nature of the topic. We conducted an initial set of think-aloud interviews with three graduate student researchers who identified with having depression and iteratively revised the protocol after each interview to clarify questions and to minimize any discomfort that the questions may cause participants. Next, we conducted a second set of think-aloud interviews with four undergraduate researchers who identified with having depression. The interview protocol was iteratively revised after each think-aloud interview until no questions were misinterpreted, and we were confident that we had minimized any discomfort that the questions may cause students (Trenor et al. 2011). A copy of the interview questions can be found in the Additional Information.

During the interview, we specifically asked students whether they revealed their depression to the individuals whom they worked with during their undergraduate research experiences; we asked about undergraduate researchers, graduate students or postdocs, and their PI or faculty mentor. We also asked students why they chose to reveal or conceal their depression. Students who revealed their identity were asked how they revealed their identity, whether they were treated differently after they revealed their identity and whether the person they revealed their identity to did anything to improve their research experience after knowing they had depression. Students who concealed their identity were asked if they would feel comfortable revealing their identity in the context of undergraduate research, if they thought they would be treated differently if they were to reveal their depression identity, and if there was anything that people in their lab could do to help with their depression. Additionally, all students were asked whether they know a scientist who identifies as having depression and what effect knowing a scientist with depression has or would have on them personally. The semi-structured nature of the interviews allowed us to explore emergent topics within a single interview that may not have been present in all interviews with students. Interviews were audio-recorded and transcribed. The interviews were approximately one hour in length. Pseudonyms were assigned to each student to protect their identity and are representative of gender. We used a gender-neutral pseudonym for the student who declined to state their gender (Jordan). Quotes were lightly edited for clarity.

### Interview analysis

First, three members of the research team (K.M.C., L.E.G., and S.E.B.) individually reviewed a different set of 10 interviews using inductive coding and took detailed analytic notes (Birks and Mills 2015). The researchers then came together to compare their notes from the interviews and discussed emergent ideas in each of the interviews. The three researchers created a preliminary codebook describing each theme. Then, two members of the research team (K.M.C. and L.E.G.) reviewed the same set of 15 randomly selected interviews to confirm the presence of the existing themes and to identify any emergent themes in the data that were missed in the initial analysis. The researchers used constant comparison methods to verify that quotes within a category were similar to one another and not too different to warrant the creation of a new theme (Glesne and Peshkin 1992). The two researchers iteratively revised their codebook until they were confident all themes that emerged from the interviews were represented and that each description of a theme adequately described the excerpts of text they had coded. Overall, the researchers analyzed the data inductively to identify themes and later compared these results with existing theory, primarily the literature related to concealable stigmatized identities (Birks and Mills 2015). Additionally, the two researchers used deductive coding to record students’ responses to questions about whether they had revealed their depression to (1) their PI or faculty research mentor, (2) at least one graduate student or postdoc in the lab, and (3) at least one undergraduate in the lab. The two researchers also recorded the number of students who reported knowing a scientist with depression. Using the final rubric, two members of the research team individually reviewed and coded a randomly selected set of the same 7 interviews (20% of all interviews). The researchers compared their codes and their Cohen’s κ interrater score was at an acceptable level (*κ* = 0.92) (Landis and Koch 1977). One researcher (L.E.G.) then coded the remaining 28 interviews. The researchers determined that there were no new themes and that data saturation had been reached with the current sample; therefore, no further recruitment was needed (Guest et al. 2006). We report all themes in the results section, each of which was reported by at least 4 students (11% of all interviewees). The final coding rubric can be found in the Additional information. Because the number of students who identified a particular theme is neither representative of the importance of the theme nor of how prevalent the theme would be within a larger population, we do not report the number of participants who reported each theme in text but do include these numbers in the Additional information. However, because we asked every student to whom they revealed their identities and whether they knew a scientist with depression, these percentages are included in the results section of the manuscript.

### Student demographics

All student demographics, including the length of time students had been in research and their year in college, were collected from the initial survey that was sent to students approximately 6 months before they were interviewed for this study.

## Results and discussion

We present the results and discussion together to elaborate further on our findings and contextualize them within the previous literature.

### Demographics

A total of 35 undergraduate researchers participated in the interviews, representing 12 of the 25 public R1 institutions that were initially surveyed. We present a summary of the participants’ gender, race/ethnicity, college generation status, transfer status, year in college, and length of research experience in Table 1.

**Table 1:** Interview participant demographics

**Participant demographics**	**Participants** ***n*= 35**
Gender	**% (*n*)**
Female	77% (27)
Male	20% (7)
Decline to state	3% (1)
Race/ethnicity	
Asian	26% (9)
Black	3% (1)
Latinx	14% (5)
Middle Eastern	3% (1)
Mixed race	3% (1)
White	49% (17)
Declined to state	3% (1)
College generation status	
First generation	29% (10)
Continuing generation	69% (24)
Declined to state	3% (1)
Transfer status	
Transfer	14% (5)
Non-transfer	83% (29)
Decline to state	3% (1)
Year in college^a^	
First year	3% (1)
Second year	14% (5)
Third year	17% (6)
Fourth year or greater	63% (22)
Decline to state	3% (1)
Length of research experience^a^	
Less than 6 months	20% (7)
6 months	17% (6)
1 year	31% (11)
1.5 years	11% (4)
2 years	6% (2)
3 years	9% (3)
3.5 years	3% (1)
Declined to state	3% (1)

^a^Students’ years in college and the lengths of their research experiences were collected using a survey that was given to students 6 months prior to the interview

### Part 1: Understanding students’ decisions to reveal or conceal their depression in undergraduate research

Studies have shown that individuals with CSIs often conceal their identities, particularly in the workplace, for fear of experiencing negative consequences (Cooper et al. 2019a; Ridge et al. 2019). Specifically, identity management of CSIs can result in strain and stress for individuals (Goffman 2009; Jones and King 2014). As such, we aimed to understand to what extent students chose to reveal their depression in research and the reasoning behind their decisions. We also explored students’ perceptions of the challenges and benefits of revealing depression in the context of undergraduate research.

#### Finding 1.1: Factors that influence students’ choices to conceal their depression in undergraduate research.

We asked students why they chose not to share their depression in the context of undergraduate research. Students recognized depression as a stigmatized identity and highlighted six reasons why they chose to conceal their depression in research.

- *Undergraduates worried that they would be treated negatively by members of the lab if they were to reveal their depression.* We explicitly asked students if they felt that they would be treated differently if others in the lab were to learn about their depression. While a subset of students did not think that they would be treated differently, students like Aaron were not sure if they would be treated differently and were unwilling to take the risk of revealing their identity to find out.

Aaron: “[I haven’t told my PI about my depression] because I think it's okay for my peers to maybe understand how I'm feeling, but I need [my PI] to have confidence that I can handle what he gives me.”

Interviewer: “And you feel as though revealing your depression to him would decrease his confidence in you?”

Aaron: “It’s hard to say, right? I can't put words in his mouth, and he’s a great guy. There’s every chance that he would still be completely supportive and wouldn’t change how he would handle the situation, but I almost can’t take that risk. You know?”

However, other students were more confident that they would be treated negatively if they were to reveal their depression to others in the lab. This aligns with the disclosure process model, which suggests that individuals who reveal their CSI are vulnerable to social evaluation. Such social evaluation can either result in greater social support or in greater stigmatization. These students describe fearing greater stigmatization if they were to reveal their CSI. Students’ primary concern seemed to be that others would assume that since they had depression, they would be less capable of handling their research-related responsibilities. Students were likely assuming that others in the lab held common negative stereotypes of people with depression, for example, that depression is a sign of personal weakness or that people with depression are unpredictable (A. H. Crisp et al. 2000; Monteith and Pettit 2011; Wang and Lai 2008), and thus they would be less capable of successfully executing their research tasks.

Interviewer: “Do you think that others in your lab would treat you differently if they knew about your depression?”

Christina: “Yeah that's kind of the fear. I don't think it would be a positive difference. I think it would be more of a negative one, where [my graduate mentor] would- I wouldn't get the same access as I do now. In the lab, the PhD student determines who gets to work on which projects and I typically work on stuff that I enjoy, but it's a little bit higher level stuff that could just as easily be given to the grad students. I'm worried that my access would be taken away a little bit if I was to do anything or if I was to say anything [about my depression].”

Many students, like Christina and Aaron, who described intentionally concealing their depression exhibited aspects of identity interference. In this case, identity interference occurred when students perceived that their depression interfered with their ability to identify as an undergraduate researcher (National Academies of Sciences, Engineering, and Medicine 2019). An identity may interfere with someone’s identity as a researcher if it threatens one’s credibility as a scientist. Specifically, Christina and Aaron both described identity interference when they worried that their mentors might perceive them as less capable of handling research-related responsibilities if they knew of their depression. Studies have shown that when students’ gender, race/ethnicity, and LGBTQ+ identities interfere with their identity as a science person, they respond by maintaining separate social and academic peer networks and compartmentalizing rather than integrating such identities with their science identity (Malone and Barabino 2009; McCoy et al. 2015; Puckett et al. 2016; Tate and Linn 2005; Yoder and Mattheis 2016). However, to our knowledge, this is the first study to demonstrate depression identity interference with students’ scientific identities, resulting in students choosing to conceal their depression.

- *Students do not have enough of a personal relationship with lab members to feel comfortable revealing their depression.* In addition to not revealing their identity in fear that they would be treated differently, some students, like Jordan, explained that they had not developed a personal enough relationship with others in the lab to reveal their depression to them.

Jordan: “I don’t have a personal relationship with [other lab members] outside of lab. Like I’ll talk to them about lab stuff, and career stuff, but I don't really talk to them about personal stuff, so [my depression] just hasn’t come up.”

Other students, such as Valerie, suggested that because they do not get along well with someone, or because they have a strained relationship, they are reluctant to share personal information with them.

Interviewer: “Would you be comfortable telling your grad student mentor about your depression?”

Valerie: “Probably not. Because I didn’t get along with her so much, I just tried to minimize the personal details I shared with her and tried to keep it very professional.”

Developing a personal relationship with someone has been widely shown to predict whether someone is willing to reveal their CSI (Chaudoir and Quinn 2010). CSI studies have found that individuals with CSIs are likely to test the waters with people, trying to predict how they would respond if they knew of their CSI (Jones and King 2014). Therefore, students, like Valerie and Jordan, may avoid sharing their identity with someone if they do not know them well and do not have an indication of how the individual may respond about their CSI.

- *Students perceive that there is no place for emotion, such as sadness or depression, in the research lab.* Students also highlighted that the science research lab is not a place for expressing emotions, feelings, or anything personal; as such, they perceived talking about depression would be inappropriate in the context of research. Justin highlighted how his mentor contributes to the perception that science is not a place for anything personal.

Justin: “I’d say [that I have not told my mentor about my depression] definitely because I feel like they act very much more professional to the level of, we don’t want to know about your personal life at all. It’s more just like we'll have lighthearted small talk but there's never any deep talking about personal experiences.”

Jacquelyn described that, interestingly, some personal information is OK to share, such as what you did on the weekend, but feelings, such as depression, are not appropriate to share with research colleagues.

Jacquelyn: “There’s just some really interesting spheres of things that seem to be in your personal life that you can share with your [research] colleagues, and then things that you don’t share. So, people talk about what they did on the weekend, but you don’t talk about your feelings really.”

The perceptions of students like Jacquelyn and Justin align with reported student perceptions of science broadly; studies have shown that students perceive that science is objective and devoid of emotion (Ebenezer and Zoller 1993; Strenta et al. 1994). The literature on emotional regulation in workplace environments suggests that individuals who perceive that there are demands to suppress unpleasant emotions, such as sadness, can experience high strain or adverse psychological, physiological and behavioral reactions to work stressors (Coté 2005). Therefore, we posit that feeling as though it is inappropriate to share one’s depression with others in research may contribute to distress in undergraduate researchers with depression.

- *Students feel that it is unnecessary to share with others in the lab that they have depression.* Other students explained that they thought it would be unnecessary or irrelevant to bring up their depression in the context of research. This is different than perceiving that it would be inappropriate to bring up their depression because emotion is not welcome in the lab; these students simply perceived that they had no reason to bring up their depression in this particular context. Students like Vanessa explained that they felt like they were doing well in research, or doing well managing their depression, so they did not see any reason to mention it to someone else in the lab.

Vanessa: “I just didn't feel like it would be necessary to tell [my mentor]. I felt like I was handling [my depression] pretty well by myself.”

Whereas students like Karla, simply did not think it was necessary for others in the lab to know about their depression.

Karla: “I don't think it's necessary for [others in my lab] to always know [about my depression].”

Studies of students with CSIs have found that students sometimes choose to reveal their CSI because they feel as though they need to get help or treatment or because it was interfering with their work (Chaudoir and Quinn 2010). Therefore, if students like Vanessa and Karla do not perceive that they need help and their depression is not interfering with their work, they may be unmotivated to share, especially if they are not seeking the social support that could potentially result from revealing their depression (Chaudoir and Fisher 2010).

- *Students report feeling uncomfortable sharing their depression.* Some students described themselves as private people who do not share much about themselves with others, or who would feel uncomfortable talking about depression in any environment, including undergraduate research experiences. For these students, it was not about the environment in which they were sharing that discouraged them from disclosing, but their personal unwillingness to discuss depression in general. Sophia explained that she views depression as something very private and highlighted that she personally might get “worked up” if she were to share about it.

Sophia: “[Depression] is something very private. So I'm not used to talking about it out in the open. This is actually the first time I've actually talked to someone about it one-on-one outside of a therapist or a psychiatric office. (...) I don't believe that there's a stigma about mental illness in the lab. I think if I were to openly talk about it, I think it would be welcome. But just personally, I can't really talk about it much without getting worked up about it.”

Similarly, Krista also described that she does not talk about her depression with anyone, regardless of whether they are her research colleagues.

Krista: “I’m not very talkative about [my depression]. Talking about it with anyone is really something I don’t do. I feel like it doesn't fit with who I present myself to other people as, if that makes sense.”

Students may not talk about their depression broadly for a number of reasons. Krista describes another example of identity interference; she perceives that her depression does not fit with who she presents herself to be. Additionally, students may experience internal stigma about their depression, especially if they learned negative stereotypes about depression early in life (Link 1987; Quinn and Earnshaw 2011; Ritsher and Phelan 2004). Therefore, these students may be even less likely to reveal their depression if they are still uncomfortable with their identity as someone with depression; such internal stigma has been shown to further exacerbate individuals’ distress regarding their CSI (Mak et al. 2007).

- *Students do not trust others to not tell other people about their depression.* Finally, some students, like Aaron and Grace, explained that they did not trust others in the lab to not tell other people about their depression or to not gossip about their depression and therefore, chose not to reveal their identity. Aaron seemed to be most concerned that other undergraduates in the lab may gossip about his depression, while Grace was concerned that she could not trust her PI to not tell others about her depression.

Aaron: “No, [no other undergraduates know about my depression]. No, because that would be a sign of weakness. I wouldn’t really want to share that. I know how easy it is to gossip about other people, and I wouldn't want to breed that.”

Grace: “From [my PIs] personality, he likes to talk and I think he’s one of those people who might let out your secret and I don’t feel like I can trust him.”

Being outed or having someone else reveal one’s CSI without their permission is an invasion of privacy and can have negative consequences on individuals with CSIs (Halwani 2002). In fact, outing students without their permission often forces students to reluctantly reveal their identities (Chaudoir and Quinn 2010). Therefore, it is logical that students like Aaron and Grace would avoid sharing their identity with anyone they could not trust to keep it a secret, subsequently protecting them from others’ potentially negative reactions.

**Fig. 1:**
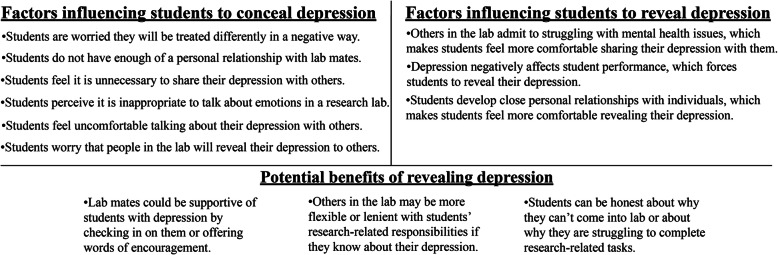
Summary of factors that influence students to conceal and reveal their depression, and the benefits of revealing depression

A summary of the factors that influenced students to conceal their depression in undergraduate research is shown in Fig. 1. Concealing one’s CSI may lead to a decreased performance in science and well-being (Quinn et al. 2014; Settles 2004). Given the potential consequences of concealing students’ depression in the context of undergraduate research, we wanted to further understand what factors helped students to reveal their depression in undergraduate research.

#### Finding 1.2: Factors that influence students to reveal their depression in undergraduate research

The subset of students who did reveal their depression to others in the lab identified three factors that helped them share their depression in undergraduate research.

- *When others in the lab admitted to struggling with mental health issues, undergraduates felt more comfortable revealing their depression.* Students described instances where others in the lab talked openly with them about their own mental health struggles. Sometimes students, such as Wendy, described experiences where they would hint about their depression or where someone would see them physically or emotionally struggling, and then their lab mate would open up about their own mental health issues.

Wendy: “When I was diagnosed [with depression] back in July, [my graduate student] was in the lab when I was having one of my breakdowns, when I was like freaking out about getting on medication. She said, ‘I'm on medication,’ which I had no idea. She goes, ‘This is a thing that happens. You take medication, you learn how to live with it, and then you just move on,’ which essentially was [the conversation] in a nutshell. It's just that it was so nice.”

Other times, students described finding out that someone else in the lab struggled with mental health, which resulted in them sharing about their depression.

Julia: “[Another undergrad and I] were waiting for a lab meeting outside of the meeting room. I was like, ‘Hey, how are you doing?’ She goes, ‘Honestly, not too great,’ so I think she was the one who brought it up first. She told me that she started on medication. I was like, ‘Oh, which one?’ It was one that I had taken before and I was like, ‘I totally know that one, I was on it for a while.’ So, we just talked about that. She asked how I was doing, and I told her.”

Students revealing their depression to others who have mental health issues echoes another study on students’ CSIs; Chaudoir and Quinn, (2014) found that a primary reason college students revealed their CSI to someone else was because that person had already revealed their own CSI. In alignment with the literature which suggests that students will try to gauge how others might respond about their CSI before revealing their own CSI (Jones and King 2014), it would make sense that students feel that they are less likely to experience a negative reaction from another person with depression.

- *Hiding depression becomes too difficult; depression affects students’ behaviors or performance and they end up having to reveal it.* Students also explained that they revealed their depression because the depressive symptoms affected their performance either in the lab or with regard to their schoolwork, and it became too difficult to hide. Interestingly, Christina and Jordan were the only two students who revealed their depression to their faculty mentor and they did so because they were no longer able to conceal it. Specifically, Christina describes that she happened to be taking a class from her PI and as such, she was unable to hide her depression when she was unable to complete an exam.

Christina: “I didn’t really have a choice. I became really depressed while I was taking [my PI’s] class and I ended up missing a midterm because I was in the hospital. I didn’t really have a choice other than to tell him what was going on because I wanted to take the midterm and not fail the class. But once I explained the gist of it, I felt really comfortable talking to him because he was so understanding in the moment.”

In Jordan’s case, their depression affected their ability to come into the lab, which ultimately led to them revealing their depression to their faculty mentor.

Jordan: “[My faculty mentor] emailed me because I hadn’t shown up to lab in two weeks, and he hadn’t really heard anything from me. He was wondering why I had missed a deadline. And I decided just to like- I met him at his office, and I just told him and I was like, ‘You know, it’s because [I have depression].’ He was like, ‘Oh, I had no idea.’ He was very supportive, and helpful with that.”

Similarly, students like Aaron and Maddie described that they revealed their depression to graduate students in the lab because it simply became too difficult to hide.

Aaron: “Yeah, [my grad student research mentor and I have] discussed [depression] before. We kind of had to discuss it because [my graduate mentor]- he has an office. I don’t. And he came into his office one day late at night, and I didn't think he would be there, and I was crying on the floor because I needed an alone space. He was like, ‘You okay?’ And I was like, ‘Yeah, I’m fine.’ So, yeah, he’s aware.”

Maddie: “Well one time that conversation [about depression] came up because I asked [my graduate mentor] to go check in on the lab because I couldn't. I was like, ‘I'm having a really rough time right now, I cannot motivate myself to go check on the crayfish, can you do that?' and he said, ‘Yes, do you need anything? Do you need to go to the counseling center on campus?’ and so on and so forth.”

Many CSIs exist on a continuum where some are completely concealable while others are more visible. While one might initially assume depression is completely inconspicuous, this finding suggests that when depression interferes with a student’s work, it can become more visible.

- *Students develop close personal relationships with others in the lab and feel comfortable revealing their depression to them.*

Students also explained that they chose to reveal their depression to others in the lab because they had developed a personal relationship with them. At times, students like Wendy described that the personal relationship minimized the chance that they would experience a negative reaction if they confided in someone about their depression. Therefore, they were more likely to share about their depression.

Wendy: “Just spending time with [another undergraduate in the lab] over the past year [caused me to tell her about my depression]. She's one of my best friends now. I definitely felt like I had that intimate relationship with her and that she would understand and that she would love me regardless. And so that was one thing that definitely made me comfortable with telling her.”

Similarly, Jordan explained how their depression became a topic of conversation once they had spent significant time talking with a graduate student in their lab.

Jordan: “I was just talking to [a graduate student] a bunch outside of lab. And then I was talking about just life things, and then it just kind of came up, and I told her about [my depression]. (…) We have weekly lab meetings. I tend to show up early, and then she asks me how I'm doing and we talk for a bit.”

The students in this study demonstrated that developing a close relationship with someone is an important precursor to revealing their CSI. We hypothesize that this close relationship may decrease the worry students have that the individual might share their CSI with others or might judge them because of their depression. Additionally, students may be receiving psychosocial support, particularly from mentors they are close with, which could indicate that they may also be a source of support given their depression.

A summary of the factors that influenced students to reveal their depression in the context of undergraduate research can be found in Fig. 1**.** Our findings about why undergraduates choose to reveal their identity in the context of undergraduate research partially align with the findings of a study of 235 undergraduate students who had a CSI, including mental health issues (Chaudoir and Quinn 2010). The study asked students about when they first revealed their identity to another person and why. Students reported revealing their CSIs because (1) they felt especially close to the person to whom they revealed, (2) the person to whom they revealed disclosed a CSI first or the participant knew that the person to whom they revealed had a CSI, (3) catharsis, or because the student wanted to get information off their chest, (4) the student was confronted with a situation and was forced to disclose, (5) the student needed to get help or treatment, or (6) the person to whom they revealed had already been told about the student’s CSI by a third party.

#### Finding 1.3: Percent of students who revealed their depression in the context of undergraduate research.

In most research institution lab settings, undergraduates are interacting with other undergraduates, graduate students, postdoctoral scholars, and a faculty member during their time in the lab. Therefore, we specifically asked each student with whom they worked and then asked whether they revealed their identity to that person. A total of 40% (*n* = 14) of all undergraduate researchers who were interviewed revealed their depression to someone in their undergraduate research lab. Undergraduate researchers were most likely to reveal their depression to other undergraduates in the lab and least likely to reveal their depression to faculty (Table 2).

**Table 2:** Percent of undergraduate researchers who revealed their depression to a lab member whom they worked with regularly

**Lab member to whom students may reveal their identity**	**Percent of participants who revealed their identity: % (*n*)**	**Percent of participants who did not reveal their identity: % (*n*)**
Another undergraduate (*n* = 30)^a^	37% (11)	63% (19)
A graduate student or postdoctoral scholar (*n* = 27)	26% (7)	74% (20)
A faculty member or PI (*n* = 30)	7% (2)	93% (28)

^a^Each *n* is based on the number of students who reported working with another undergraduate, a graduate student or postdoc, or a PI in their undergraduate research experience

Why were undergraduates most likely to reveal their depression to other undergraduates and least likely to reveal their depression to a faculty member? The two students who revealed their depression to a faculty member did so because their depression interfered with their performance in the faculty member’s class or in the research lab. Conversely, students primarily chose to reveal their depression to other undergraduates and graduate students because of their close relationships with them. Students may not often have the opportunity to build the type of relationship with faculty that is needed to feel comfortable revealing their depression. Since students at research-intensive institutions are more likely to interact with postgraduate mentors than with faculty (Aikens et al. 2016), we posit that postgraduate mentors, including both graduate students and postdocs, are in a unique position. They may be able to cultivate strong relationships with undergraduates that facilitate the potential revelation of a students’ depression, even though they are in a position of power.

#### Finding 1.4: Benefits of revealing depression and the challenge of concealing depression.

We identified a set of three potential benefits associated with revealing one’s depression in undergraduate research. These benefits were derived from reports of students who had revealed their depression to others in the lab. However, the same themes were echoed by students who had not revealed their depression; these students hypothesized that they would benefit in the same ways. To fully describe the theme, we chose to include quotes from students who had experienced the benefits and from students who hypothesized about the benefits. Additionally, we identified a single challenge of concealing depression: students who are not out about their depression cannot be honest about why they are not able to complete research-related tasks. We discuss this challenge at the end of this section.

- *Benefit of revealing depression: Students could receive support from lab mates if they revealed their depression.* Students who had revealed their depression to others in the lab, especially to graduate students, described receiving support from them. That is, graduate students would check in on them or ask how they were doing.

Jordan: “[The one grad student who knows about my depression], she asks me how I'm doing. And that's definitely very helpful to have someone who, on a weekly basis, wants to talk to me and wants to make sure that I'm doing okay.”

Students who had not told others in the lab about their depression perceived that revealing their depression could result in others in the lab providing support or checking in on them.

Katherine: “If I were to tell [my graduate mentor about my depression] she would probably ask if there's anything she can do and if there’s anything about the lab that's exacerbating it, because I'm sure she would strive to turn it around.”

Other students envisioned that they might receive support in the form of their graduate student mentor providing words of encouragement or sharing similar experiences regarding mental health.

Illana: “[If my graduate student mentor knew about my depression] maybe they’d just give me words of encouragement. Or maybe share their own experience if they are going through the same thing, what they did to help them cope.”

Interviewer: “Why are words of encouragement helpful, particularly when you're going through a depressive episode?”

Illana: “Because when I'm going through a depressive episode, I’m always telling myself that I can't do it. So, I'm kind of bringing myself down. And, even when I try to bring myself up, it just means nothing. And then, when someone else says it, I just feel, like if they truly mean it, and I'm assuming they know me as a person, then they'll be telling me, ‘I know you're capable of this. I know you can do it.’”

The disclosure process model suggests that if individuals disclose information to others about their CSI, then it can change their social support by resulting in greater social support or greater stigmatization. Students, like Illana and Katherine, highlighted that they perceived that their social support structure would change in a positive way if they revealed their depression in research. By revealing their depression, they perceived they would gain social support in the form of others checking in on them and encouraging them.

- *Benefit of revealing depression: Others in the lab may be more flexible or lenient.* Some students who had revealed their depression to their mentors described that their mentor changed their behavior toward them after the mentor knew about their depression. Specifically, students mentioned mentors being more flexible with deadlines or with students’ abilities to complete specific tasks.

Olivia: “I was on the microscope counting cells and it was like really repetitive work. I was losing track of which samples I had already counted and was getting frustrated. Halfway through the tasks, [my postdoc mentor] was like, ‘If this is stressful, or you're getting frustrated, feel free to go home.’ I’m so glad that I was able to say, ‘I’m just having a bad day. I need to go home.’ And her knowing the backstory [about my depression], she was able to know that, that’s what it was and that I could step away and I’ll come back tomorrow and be fine.”

Other students who had not revealed their depression to others in the lab imagined that revealing depression could result in their research mentors being more flexible or understanding.

Vanessa: “I feel like [the grad student] wouldn't end up treating me differently [if she knew about my depression] in like, her feeling weird about me. But maybe being more lenient and trying to accommodate me better. For example, we have schedules to feed the snakes and weigh them. I feel like maybe if she knew [about my depression], she would come to me first, to let me pick first, and then let everyone else pick and choose the schedule they want.”

Vanessa went onto explain that with her depression it could be especially hard to come in on certain days when she was already experiencing a lot of stress, so having choice over her time would have helped her.

The disclosure process model also highlights that when an individual reveals their CSI, it can change their social information; that is, their CSI is now “out in the open” and can change the perceptions and actions of both the individual who reveals the CSI and the individual whom it is revealed to. In other words, confidants now have new information about the disclosers and the disclosers are aware of that. Vanessa and Oliva describe that revealing their depression changed their social information such that once another person in the lab knew about their depression, the other person’s behavior changed by becoming more flexible and lenient.

- *Benefit of revealing depression: Students could be honest about why they cannot come into lab or why they are struggling to complete a task.* Students described that the benefit of being out to their mentors about their depression was that they could be honest about when they were struggling to complete a research-related task because of their depression. In some instances, like Maddie’s, it resulted in their graduate mentor offering additional help with research tasks.

Maddie: “Yes, it is [helpful that my grad mentor knows about my depression], because there have definitely been times where I’ve called and said ‘I'm having some really bad mental health days, will you please go in and just feed them and check on [the crayfish]?’ And he's like, ‘Yes of course. Do you need anything? Is there anything else I can do?’”

In these cases, the disclosure process model highlights that disclosing students’ identities allowed for their own behavior to change. They can now be more honest about why they cannot come into the lab or complete a task on days when they felt depressed.

- *Challenge of concealing depression: Students who are not out about their depression are not able to be honest about when they are struggling to complete a task.* Conversely, students who were not out about their depression cited that a major challenge of concealing one’s depression was not being able to be honest about when they are struggling to complete a task. Students imagined that it could be helpful if others knew about their depression because when they were unable to perform to the best of their ability, they could explain that it was because of the depression, and not because they were intellectually incapable of completing a particular task.

Jennifer: “I’ve turned in presentations to [my research mentors] that are not professional or polished. It's kind of glaringly obvious if it’s something that I haven’t completed. They definitely notice. [It would be helpful if they knew about my depression] because I could present it as, ‘Hey, this is what I was able to accomplish with the energy levels and motivation levels. This is what I have for you.’ Rather than falling short of what they’re asking me and them not knowing why I wasn’t able to complete what they set forth.”

Michael: “If you were just, say, sick with some bodily illness, you could be totally honest and say, ‘Hey, I’m not feeling good today. I cannot come in.’ But since it’s a mental illness that’s stigmatized, you can’t quite be honest. You just have to say, ‘Oh, I have a lot going on.’ You have to be very vague (…) I don’t feel comfortable doing that. So, a lot of times, I just bite the bullet and go in but yeah, it’d be nice if I could just say, ‘Is it cool if I come in late or if I don’t come in at all?’ That’d be nice.”

Given the stress that concealing a CSI can have on students, the potential benefits of revealing depression, and our findings highlighting that students’ relationships with others in the lab can help them feel more comfortable revealing their identities, we recommend that individuals in lab leadership positions, including graduate students, postdocs, and faculty members, take the time to develop relationships with undergraduates. While forming positive relationships with mentors has been shown to positively impact student persistence in undergraduate research experiences (Cooper et al. 2019b), we hypothesize that developing such relationships will help make students with depression more comfortable in the research environment. Additionally, explicitly working to destigmatize mental health issues may also positively affect students; reminding lab members that mental health issues are common can provide students with a cue that lab members are understanding of mental health issues and may encourage undergraduates with depression to reveal their identity, allowing for better mentorship.

### Part 2: Undergraduate researchers’ perceptions of scientists with depression

In addition to understanding the motivation behind students’ decisions about whether to reveal their depression in undergraduate research, we were also interested in learning whether students knew any scientists with depression, and how, if at all, knowing a scientist with depression affected them or could affect them.

#### Finding 2.1: Likelihood that students know a scientist with depression.

Only 11 students (31%) said that they knew a specific scientist with depression. In the interview question asking students whether they knew a scientist with depression, we did not specify that students needed to know the scientist personally, but all of the students who said that they did know a scientist with depression referenced a personal relationship. Of the 11 students who said that they knew a scientist with depression, five students said that the scientist whom they knew with depression was a graduate student and one said that the scientist with depression whom they knew was a postdoc; therefore, only five students (14% of all students we interviewed) knew a practicing scientist who had depression who was not still in training (Table 3).

**Table 3:** The percent of participants who reported knowing a scientist with depression

	% (*n*)
	*n* = 35
Students who knew a scientist with depression	31% (11)
Students who knew a practicing scientist with depression	14% (5)
Students who knew a postdoc with depression	3% (1)
Students who knew a graduate student with depression	14% (5)

Why do so few students know scientists with depression? Depression is a stigmatized identity in the sciences, and although the scientific community is beginning to recognize the potential prevalence of depression and the importance of supporting science graduate students with depression (Flaherty 2018; Woolston 2019), it is possible that others in the academic community may not respond positively if a scientist were to reveal their depression (Jago 2002). As with any CSI, scientists who shared that they experience depression would be taking a risk that others would judge them for having a CSI (Quinn and Chaudoir 2009). However, we also hypothesize that scientists may not realize how revealing their depression could positively impact students. In a study exploring biology instructor decisions to reveal their LGBTQ+ identity to students in their classrooms, researchers found that even if instructors perceived no costs to revealing their CSI, they still concealed their identity unless they thought revealing it would benefit students (Cooper et al. 2019a). When instructors were questioned about how revealing their LGBTQ+ identity may affect LGBTQ+ undergraduates with the same identity, many perceived it could have a positive effect, but admitted that they had never thought before about the impact that revealing their LGBTQ+ identity would have on students. Therefore, scientists with depression may not have considered how revealing their identity may positively affect undergraduates with depression.

#### Finding 2.2: The potential benefits of knowing a scientist with depression.

We asked students who did not know a scientist with depression how they might be affected if they did know a scientist with depression. Students described that knowing a scientist with depression could help them in two ways: it could demonstrate that people with depression can be successful in science and it can help a student realize that they are not the only one experiencing depression in science.

- *Knowing a scientist with depression could demonstrate that people with depression can be successful in science.* Students, like Wendy, Tim, and Jennifer, described that knowing someone with depression would show that people with depression can still be successful in science.

Wendy: “[Knowing a scientist with depression] would definitely, be like a beacon in the community. It would show that someone can be very successful despite what they face. They’re successful even though they have invisible challenges and people don't believe that there is such a thing as depression. They still manage to make it through. So that would be, that’d be amazing if we had more people identify as suffering from depression either currently or in the past.”

Tim: “Having somebody else [who is a scientist with depression] tell me, ‘Hey. I am successful, and I still suffer from depression.’ I’m not going to say, ‘Oh, thank god I’m not the only one’, because I don’t want anybody else to suffer, but knowing that you can succeed even with this extremely difficult thing weighing you down would help a lot because it tells me that this is not a pointless endeavor. It’s not a zero-sum game. I’m not going to just screw this up and fail because other people have managed to do it too. I'm not the only one who’s fighting this battle.”

Jennifer: “[Knowing a scientist with depression] would definitely make me feel more capable. It would be someone I could look to, ‘Hey they're dealing with the same things that I do, and they're still able to hold this position, and get research done. They published all this stuff.’ It would be a role model for me, to be able to look up to them, ‘Hey, if they can do that, and deal with all the stuff they've been dealing with, I can deal with it too.’”

Individuals with depression are often self-critical (Blatt et al. 1982; Gilbert et al. 2006) and may be more pessimistic about their ability to be successful in science than individuals who do not have depression (Cane and Gotlib 1985). Therefore, having a scientist with depression who demonstrates that it is possible to be successful in science may be particularly impactful for undergraduates with depression.

- *Knowing a scientist would help students realize that they aren’t the only one experiencing depression in science and could provide someone to talk with about depression.* Finally, on a more personal note, students, like Nancy and Christina, highlighted that knowing someone with depression could help them realize that they are not the only person in science with depression.

Nancy: “[Knowing a scientist with depression] is good, it’s good because you know you're not alone. Everyone's sad and you're working through it together, so it’s a community.”

Christina: “I think it would be really helpful [seeing scientists with depression] to kind of see that you're not alone and like there are other people that are trying to deal with very similar or the same things that you are. Just having that ability to see someone that's like you is super helpful in just general life but also in research.”

Additionally, students like Matt, highlighted that knowing a scientist with depression could provide them with someone to talk with about both science and depression.

Matt: “I think it would probably help me discuss [my depression] with somebody who shares a lot of common ground in what we [as science people] deal with on a daily basis.”

Knowing other scientists with shared experiences and feeling less alone, may provide students with a sense of belonging within the scientific community (Cooper and Brownell 2016).

Previous studies have identified that having mentors with similar identities can benefit students, particularly in undergraduate research (Blake-Beard et al. 2011; Emery et al. 2019; Felder and Barker 2013; Hurtado et al. 2009). This is the first study, to our knowledge, to highlight that individuals revealing depression can serve as role models to others with depression who look up to them; more specifically, we identified that mentors revealing depression may positively affect undergraduate researchers. Scientists may be more willing to reveal this CSI if they knew of these possible ways that students could benefit from knowing scientists with depression. We recognize that not all mentors struggle with mental health issues and thus, cannot act as role models for students with depression. However, simply helping students know about other scientists with CSIs may benefit students (Barnes and Brownell 2017; Schinske et al. 2016). For example, informing students that there have been a number of successful scientists, such as Charles Darwin and Buzz Aldrin, who were thought to have had depression and other mental health issues (Lee 2009; Lehrer 2010) may help students with depression feel as though they belong more to the scientific community.

### Limitations

The students with depression who participated in interviews for this study volunteered to do so. Therefore, these students may be more comfortable with their depression identity than the average student and thus we may be overestimating the students who are willing to reveal their CSI in scientific research. Additionally, students’ willingness to reveal their depression and their reasoning as to why they would reveal their depression may be dependent on the time that students have spent in research. For example, six of the seven students who had been in research for at least 2 years had revealed their depression to at least one other person in their lab, often a graduate student or postdoctoral scholar. Students may be more likely to reveal their depression later in their research experience because they become more comfortable or because they are more likely to encounter a situation where they are forced to; more research is needed to fully understand the relationship between time in research and revealing of CSIs. Additionally, all students were recruited from public R1 institutions; therefore, these results cannot be generalized to other institution types (e.g., community colleges, primarily undergraduate institutions, master’s granting institutions). Finally, we did not explicitly explore the challenges of revealing depression or the benefits of concealing depression; however, many potential challenges related to revealing depression were present in the reasons students chose to conceal their depression, as were the protective benefits of concealing depression.

## Conclusion

In part 1 of this study, we explored to what extent students with depression revealed their concealable stigmatized identity or CSI (depression) in undergraduate research and the decision-making processes behind revealing or concealing their depression. We found that most of the students did not reveal their depression to others in undergraduate research, but those who did were most likely to reveal it to another undergraduate researcher and least likely to reveal it to a faculty mentor. Students concealed their depression because they feared that others would treat them negatively and limit their research-related responsibilities, because they perceived that emotion is not welcome in science, because they were uncomfortable sharing their depression, or because they did not perceive revealing depression to be relevant to their undergraduate research experience. Students revealed their depression because they were close to someone in the lab, because another lab member also experienced mental health issues, or because they experienced depression-related challenges that forced them to reveal their identity. Further, we identified a suite of benefits students experienced once they reveal their depression, including support and flexibility from research mentors. In part 2 of this study, we explored whether students knew a scientist with depression and whether they perceived knowing a scientist with depression could benefit them. We found that very few students knew a scientist with depression. However, students identified that if they did know scientists with depression it would help them realize that people with depression can be successful in science and help students with depression feel less alone in the scientific community. We hope that this work encourages undergraduate research mentors to be supportive of students with depression and to work to lessen the stigma surrounding this CSI. If you or someone you know experiences depression and would like help, we have provided a list of resources in the Additional Information.

## Supplementary information

**Additional file 1.**

## Data Availability

Underlying data are subject to ethical restrictions as the interview transcripts contain identifiable information, including student names, the names of PIs and graduate and undergraduate researchers, and anecdotes that could identify the students. Further, the interviews include sensitive information about students’ mental health and students were assured before their interview that their transcripts would never be shared with anyone outside of the research team. It is for these reasons that the data will not be shared. However, the interview protocol is available in the Additional Information.

## Abbreviations

CSI: Concealable stigmatized identity
STEM: Science technology engineering and mathematics
U.S.: United States of America

## Footnotes

1 Gender and race/ethnicity are often categorized as conspicuous identities because they tend to be immediately visible. However, we recognize that this is not always the case; for some people, their outward appearance does not align with the gender or race/ethnicity that they identify as.
