# Supplementary material for: Depression as a concealable stigmatized identity: what influences whether students conceal or reveal their depression in undergraduate research experiences?
Source: Int J STEM Educ. 2020 Jun 4;7(1):27. doi: 10.1186/s40594-020-00216-5 (PMC7271012; doi:10.1186/s40594-020-00216-5)
Supplement: Supplementary file 1 — Additional file 1. [file 40594_2020_216_MOESM1_ESM.docx]

Additional information for

***Depression as a concealable stigmatized identity: What influences whether students conceal or reveal their depression in undergraduate research experiences?***

This supplement contains the following:

Item Page

Copy of interview questions analyzed 2

Copy of student demographic questions 4

Copy of final coding rubrics and the number of students who reported each theme 6

Resources for individuals with depression 11

**Copy of interview questions analyzed**

The goal of this study is to interview students about their experiences with depression in the context of undergraduate research experiences, so my first question is, have you experienced depression, either currently or in the past?

*Part I: Relationships in the lab and depression*

Have you worked with a **graduate student or postdoc** research advisor during your undergraduate research experience?

- If yes:
  - Does your **grad student or postdoc** research advisor(s) know about your depression?
    - If yes:
      - How did they find out?
      - Did you feel that they treated you differently after they knew about your depression?
      - Is there anything that your research advisor did to improve your experience in undergraduate research, given your feelings of depression?
      - Do you think there is anything they could do to help you, given your depression?
    - If no:
      - Why not?
      - Would you have felt comfortable telling them?
        - If yes:

Why did you not reveal your depression to them?

- - - - Do you think they would have treated you differently if they knew about your depression?
      - If they did know about your depression, do you think there is anything they could do to help you, given your depression?

Have you worked with your **faculty research advisor** during your undergraduate research experience?

- If yes:
  - Does your **faculty research advisor** research advisor know about your depression?
    - If yes:
      - How did they find out?
      - Did you feel that they treated you differently after they knew about your depression?
      - Is there anything that your faculty research advisor did to improve your experience in undergraduate research, given your feelings of depression?
      - Do you think there is anything they could do to help you, given your depression?
    - If no:
      - Why not?
      - Would you have felt comfortable telling them?
        - If yes:

Why did you not reveal your depression to them?

- - - - Do you think they would have treated you differently if they knew about your depression?
      - If they did know about your depression, do you think there is anything they could do to help you, given your depression?

Have you worked with any undergraduate researchers during your undergraduate research experience?

- If yes:
  - Do any of the **undergraduate researchers** in your lab know about your depression?
    - If yes:
      - How did they find out?
      - Did you feel that they treated you differently after they knew about your depression?
      - Is there anything that undergraduates in your lab did to improve your experience in undergraduate research, given your feelings of depression?
      - Do you think there is anything they could do to help you, given your depression?
    - If no:
      - Why not?
      - Would you have felt comfortable telling them?
        - If yes:

Why did you not reveal your depression to them?

- - - - Do you think they would have treated you differently if they knew about your depression?
      - If they did know, do you think there is anything they could do to help you, given your depression?

*Part II: Role models*

Do you know anyone with depression who is a scientist?

- If no:
  - How would knowing a scientist with depression affect you?
- If yes:
  - How has knowing a scientist with depression affected you?

**Copy of student demographic questions**

I most closely identify as

- Female
- Male
- Other (please describe)
- Decline to state

I most closely identify as

- American Indian or Alaska Native
- Asian
- Black or African American
- Hispanic, Latino, or Spanish origin
- Pacific Islander
- White/Caucasian
- Other (please describe)
- Decline to state

I most closely identify as

- A first-generation college student whose parents’ highest level of education is a high school diploma or less
- A first-generation college student (at least one parent has some college)
- Non-first-generation college student (at least one parent has finished college)
- Decline to state

Please indicate the option that most closely reflects your college experience

- I transferred to this institution from a 2-year college, community or junior college
- I transferred to this institution from another 4-year institution
- I started my college career at this institution
- If none of the above reflect your experience, please describe your experience below
- Decline to state

How long have you attended college while pursuing your undergraduate degree?

- 1 year or less (first-year student)
- 2 years (sophomore)
- 3 years (junior)
- 4 years (senior)
- 5 years or more
- I have graduated with my undergraduate degree
- Decline to state

Please choose the response that reflects how long you participated in your undergraduate research experience

- Less than 6 months
- 6 months
- 1 year
- 1.5 years
- 2 years
- 2.5 years
- 3 years
- 3.5 years
- 4 years or more
- Decline to state

**Rubric 1: Reasons why students choose not to share their depression with others in undergraduate research. (n = 35)**

| **Theme** | **Description of the theme** | **n (%)** |
| --- | --- | --- |
| Don't want to be treated differently | Student acknowledges that they think it would be possible they would be treated differently in a negative way if they were to reveal their depression to others in their research lab. This includes students who don’t know but avoid sharing their identity, just in case they might be treated differently. | 18 (51%) |
| Do not have a personal relationship with them | Student describes that they do not have a personal enough relationship with someone in their lab to tell them that they have depression. | 15 (43%) |
| Unnecessary to share that information | Student describes that it is unnecessary to share the information because it is irrelevant to a work environment. | 11 (31%) |
| Research is not a place for emotions | Student describes that they perceive there is no place for feelings such as depression in the lab. They may also describe that it is inappropriate or unnatural to share feelings or talk about personal things in the workplace. | 10 (29%) |
| Personal reason for not sharing (e.g. it would make them upset) | Student describes that they would feel uncomfortable sharing that part of themselves or they emotionally struggle to open up about their depression to someone else. | 5 (14%) |
| Can't trust someone to keep your secret | Student describes that they would not want to share that they have depression because the person may tell others in the lab. | 5 (14%) |

**Rubric 2: Reasons why students choose to share their depression with others in undergraduate research. (n=35)**

| **Theme** | **Description of the theme** | **n (%)** |
| --- | --- | --- |
| Others in the lab admit to struggling with mental health issues too | Student describes that they have revealed their depression to someone who has been affected by mental health issues. This could be that they themselves experience a mental health issue or that they are close with someone who experiences a mental health issue. | 7 (20%) |
| Hiding depression becomes too difficult because it affects work or behavior | Student describes that they revealed their depression because they essentially had to. Often students describe that their depressive symptoms affected their work (e.g. missing deadlines, not showing up), which ultimately led to them revealing their depression to someone in the lab. | 5 (14%) |
| Student has developed a close personal relationship with others | Student describes that they are willing to share their depression with someone because they have a close personal relationship with them. | 4 (11%) |

**Rubric 3: Potential challenge of student concealing depression in the context of undergraduate research. (n = 35)**

| **Theme** | **Description of the theme** | **n (%)** |
| --- | --- | --- |
| Cannot truthfully explain why they are unable to complete a task or come into lab | Student describes that one challenge associated with not revealing their depression in undergraduate research is that they are not able to explain why they are unable to come into lab some days, since nobody knows about their depression. | 18 (51%) |

**Rubric 4: Potential benefits of student revealing depression in the context of undergraduate research. (n = 35)**

| **Theme** | **Description of the theme** | **n (%)** |
| --- | --- | --- |
| Could receive support from lab mates | Student describes they may receive support and understanding from others who know about their depression. Students describe that mentors may check in on them from time to time or offer them words of encouragement. | 23 (66%) |
| Others in the lab may be more flexible or lenient | Student describes that if someone knows of their depression they may be more flexible with their schedule or more lenient with deadlines, etc... | 11 (31%) |
| Student can be honest about why they can't come in/complete a task | Student describes that being out about their depression would allow them to be honest with others in the lab about why they cannot go into lab or complete a task. | 6 (17%) |

**Rubric 5: Reasons why students perceive it would be beneficial to know someone in science with depression. (n = 35)**

| **Theme** | **Description of the theme** | **n (%)** |
| --- | --- | --- |
| Example that student can be successful with depression | Student describes that knowing a scientist with depression provides an example that people can be successful in science even if they have depression. | 12 (34%) |
| Knowing student is not alone | Student describes that knowing a scientist or knowing of a scientist with depression helps or would help them realize they're not alone as someone with depression in science. Student may also describe that knowing a scientist with depression would provide them with someone to talk with about their experience having depression in science. | 12 (34%) |

**Resources for individuals with depression**

Web resources:

- Anxiety and Depression Association of American website: <https://adaa.org/>
- Depression and Bipolar Support alliance: <http://dbsalliance.org>
- MentalHealth.gov website: <https://www.mentalhealth.gov/>

24/7 free services and crisis hotlines:

- Crisis Text Line, which allows you to text a trained live crisis counselor (Text “CONNECT” to 741741)
- National Suicide Prevention Lifeline at 1-800-273-8255 (“TALK”)
- Disaster Distress Helpline: Call 1-800-985-5990 or text “TalkWithUs” to 66746

Many universities also offer counseling centers and university-dedicated hotlines. For example:

- ASU Counseling Services: <https://eoss.asu.edu/counseling>
- ASU 24-hour crisis hotline: 480-921-1006
- ASU crisis text line: Text “HOME” to 741741
